# Features of Adolescents Tuberculosis at a Referral TB’s Hospital in Tehran, Iran

**DOI:** 10.4084/MJHID.2016.005

**Published:** 2016-01-01

**Authors:** Ferial Lotfian, Mohammad Reza Bolursaz, Soheila Khalilzadeh, Noshin Baghaie, Maryam Hassanzad, Aliakbar Velayati

**Affiliations:** 1 Clinical Tuberculosis and Epidemiology Research Center, National Research Institute of Tuberculosis and Lung Diseases (NRITLD), Masih Daneshvari Hospital, Shahid Beheshti University of Medical Sciences, Tehran, IR Iran.; 2 Pediatric Respiratory Diseases Research Center, National Research Institute of Tuberculosis and Lung Diseases (NRITLD), Masih Daneshvari Hospital, Shahid Beheshti University of Medical Sciences, Tehran, IR Iran.

## Abstract

**Objective:**

To identify the pattern of the clinical, radiological, diagnostic procedures and loss to follow-up of the diagnosed cases of active tuberculosis (TB) adolescents.

**Methods:**

This study was a retrospective analysis of the medical records of 143 adolescents aged 10 to 18 years with tuberculosis who were admitted TB wards of National Research Institute of Tuberculosis and Lung Disease (NRITLD) in Tehran, Iran, between March 2006 and March 2011.

**Results:**

Of the 143 patients identified, 62.9% were females. Median age of the patients was 16 years. The contact source was identified in 47.5%. The most common presenting symptom was cough (86%). Isolated pulmonary TB (PTB) was detected in 113 patients (79%), 21 patients (14.7%) had extrapulmonary TB(EPTB), and 9 patients (6.3%) had PTB and EPTB. The most common site of EPTB was pleural (14%). The most common radiographic finding was infiltration (61%). Positive acid fast smears were seen in 67.*6*%. Positive cultures for *Mycobacterium tuberculosis* (*M. TB*) were seen in *44.7*%. Positive Polymerase chain reaction (PCR) results were seen in 60%. The adolescents aged 15 to 18 years were more likely to lose weight (p=0.001), smear positive (p=0.001), culture positive (p<0.001) and have positive PCR results (p=0.009). The type of TB (p=0.017) was a significant factor influencing loss to follow-up.

**Conclusions:**

The study has revealed that the clinical and radiological findings of TB in adolescents are combination as identified in children and adults. The TB control programs should pay more attention to prevention and treatment of TB in adolescents.

## Introduction

Tuberculosis (TB) remains a major health problem globally with 8.6 million people identified in 2012 of whom 1.3 million died.^1^ Recent data from Iran estimated the incidence rate of TB as 21 cases per 100,000 population in 2012.^2^ According to a recent study in South Africa, among 29,478 newly notified TB cases, the incidence rate started to peak in adolescents.^3^ Recent studies have shown that adolescents are a susceptible age group with a higher case incidence compared to young children.^4^–^6^ There are few studies on adolescent TB in the literature and reports indicate that many adolescents with active TB are diagnosed during the late stage of the disease.^7^ One study performed in Canada showed that the average interval between the beginning of symptoms and diagnosis of TB was 5 months.^8^ The delay in the treatment due to delay in diagnosis of TB leads to increase in the infectivity of the infected person in the community as a result of social interactions in this age group. The aim of this study was to evaluate the demographic data, clinical presentations, radiologic features, microbiological findings, site of TB, treatment, and outcome in this age group.

## Material and Methods

This study was conducted on all patients aged 10 to 18 years old with a confirmed diagnosis of TB, who were admitted to TB wards of National Research Institute of Tuberculosis and Lung Disease (NRITLD) in Masih Daneshvari Medical Center between March 2006 and March 2011. NRITLD is a World Health Organization (WHO) Cooperative health center for TB and lung disease, located in Tehran, Iran. NRITLD provides particular care for TB patients who referred from across the country. As in WHO definition, adolescents group was defined as any person between ages 10 and 18.^9^ The following data was analyzed in this study: demographic data, presenting symptoms, radiographic features, bacteriological results, tuberculin skin test, and treatment, outcome, and drug susceptibility results. Patients with TB disease were defined in two categories: 1) Definite TB was defined by identification of Mycobacterium tuberculosis (M. TB) from the sputum, gastric lavage, body secretions, or surgical specimens, or the histological appearance of biopsy material representing TB-affected tissues (caseous necrosis or granulomatous tubercles). 2) Probable TB was referred as the presence of 3 or more of the following: symptoms and signs consistent with active TB; abnormal radiography of TB (hilar lymphadenopathy and/or consolidation); history of TB contact and either response to TB therapy. Then, cases were divided into two age groups, 10–14 and 15–18 years.

Tuberculin skin test (TST) and radiological findings could not be determined for all patients through the information in the medical files since the study was retrospective. TB treatment has been started at the time of diagnosis, as recommended by the WHO guidelines, consisting of isoniazid, rifampin, ethambutol and pyrazinamide for an initial 2- month phase followed by isoniazid and rifampin for a maintenance 4-month phase.^10^

SPSS version 21 was used for data analysis. The Chi-square or Fisher’s exact test was used to evaluate the level of significance. The Mann-Whitney and Kruskal-Wallis were used to compare the relationship between an increase in age and positive acid-fast smears, and increase in age and cavitary lesions, respectively. A p-value less than 0.05 was considered significant.

## Results

We Identified 143 patients with TB, 115 (80.4%) definite TB and 28 (19.6%) probable TB (Table 1). Ninety (62.9%) were female, and 53 (37.1%) were male. The median age was 16 years. Of these, 79 (55.2%) were Iranian and 64 (44.8%) Afghan. Scar of Bacille Calmette-Guerin (BCG) vaccine injection was seen in 27(19%), in 10(7%) not seen the scar and in 106(74%) the scar was unknown. The contact source was identified in 68cases (47.5%); 24 (60%) in 10–14 years and 44 (42.7%) in 15–18 years old. The difference between two age groups is described in Table 2. Common contact sources were parents 26(18.2%). HIV was only tested for 56 out of 143 patients, among them two patients (3.5%) were HIV positive and presented with pulmonary localization.

A TST result was available in 61 patients. Of these, Thirty-two (22.3%) patients had induration that exceeded 10mm. There was no significant difference in TST positivity between two groups. (p-value: 0.167) (Table 2). However, there may have been some selection bias regarding whose results were available for TST, making a difference in TST positivity between the groups.

Most patients 138 (96.5%) were recognized by presenting symptomatically. The most common symptoms were a cough 123 (86%), fever 104 (72.7%) and weight loss 94 (65.7%). The median of symptoms duration was two months. Five patients were asymptomatic at presentation; all of them had the history of contact, reactive TST and pulmonary involvement in chest radiographic.

Isolated pulmonary disease occurred in 113 (79%), 21 (14.7%) had extrapulmonary disease, and 9 (6.3%) had pulmonary TB(PTB) and extrapulmonary TB(EPTB); EPTB distributed as pleural 20 (14%), central nervous system (CNS) 4 (2.8%), lymph node 1 (0.7%), pleural with peritoneal 2 (1.4%), pleural with skeletal 1 (0.7%), and CNS with lymph node 1 (0.7%).

**Figure 1:**
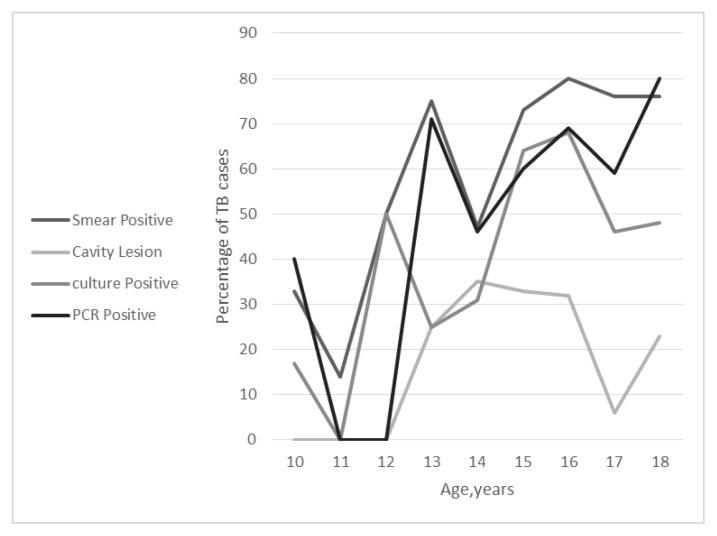
Age distribution of adolescents with smear positivity, culture positivity, PCR positivity and with the presence of cavity lesion.

Positive Acid-fast smears were seen in 96 of 142 patients (67.6%). The most common site of smear positive was sputum 76 (53%). The smear-positive rate increased with age (33% at ten years to 76% at 18 years) (Mann-Whitney: p=0.003) peak age was 16 years (80%) (Figure 1). Cultures were achieved in 123 patients. Positive cultures for M. TB were seen in 55 (44.7%); fifty patients with pulmonary disease, one patient with EP TB and four patients with both disease. Culture-positive rate increased with age (17% at 10 years to 48% at 18 years) (Mann-Whitney: p=0.039) (Figure 1). Polymerase chain reaction (PCR) test of M. TB were performed for 88 patients; positive PCR was seen in 52 patients (60%). The common site of positive PCR test was sputum in 38 patients (73%). PCR-positive rate increased with age (40% at 10 years to 80% at 18 years) (Mann-Whitney: p=0.018) (Figure 1).

Twenty-one Patients were confirmed as TB cases by a histological diagnosis (pleural n=14, bronchial n=6, lymph nodes n=2, bone n=1). Drug susceptibility testing (DST) for M. TB was performed in 15 patients. Seven patients had susceptible M. TB strains to all anti-TB drugs, eight patients had isolates of M. TB drug-resistant: 5 had multidrug-resistant TB (three with resistance to rifampin and isoniazid, another with additional resistance to ethambutol, amikacin, and kanamycin), three patients had drug resistance to isoniazid and one to pyrazinamide.

Radiography reports were available in 107 patients; 23 cases had chest X-ray, and 84 cases had CT scan reports. Abnormalities were found in 102 (95.3%). Of these patients, 62 of 102 (61%) had infiltration, 52 of 102 (51%) had consolidation, 30 (29%) cavity, 25 (24.5%) pleural effusion and 23 (22.5%) adenopathy. Cavitary lesion was increased with age (0 at ten years to 23% at 18 years) but the difference is not statistically significant (by Kruskalwallis: p=0.373) (Figure 1)

Eighty-nine patients (61%) had complete follow-up; 71 (49.7%) improvement, 14 (9.8%) relapse, 3 (2.1%) expire and 1 (0.7%) sequelae. Fifty-four (39%) adolescents identified as a loss to follow-up TB treatment. The comparison between adolescents loss to follow–up and not were performed to found if any factors based on our research that influenced loss to follow –up. A significant statistical difference was found for type TB (p=0.017) between adolescents identified as a loss to follow –up and not (Table 3).

Elevation of AST and ALT enzymes occurred in 9 of 143 patients (6.2%); seven patients were between15 and 18 years. However, restored to the normal range without any disruption of treatment.

## Discussion

This study reports the epidemiology and characteristics of TB in adolescents from a developing country. To our knowledge, no similar study has been reported from other Middle East countries. Clinical and radiological features of adolescents TB are different from children and adults. Older adolescents had a severity of TB disease than younger adolescents.

Adolescents are more frequently symptomatic.^8^, ^11^, ^12^ We also found most patients (96.5%) were recognized after presenting with symptoms. Isolated PTB and EPTB were detected in 79.7% and 14.7% of the patients in this study, respectively. Previous studies detected PTB and EPTB in 22% to 78.6% and 17% to 35% of the adolescents with TB, respectively.^8^, ^11^, ^12^ The proportion of patients with EPTB compared with those of PTB varies among countries and depends on host-related factors such as ethnicity, and associated diseases.^13^ In this study, the frequency of EPTB was closer to the rate in adults (16%) as compared to children (27.3%).^14^ The most common form of EPTB in our adolescents was pleural, similar to studies conducted in France and Canada.^8^,^12^ This is in contrast to studies conducted in the USA and South Africa that found the most common site of EPTB was peripheral lymphadenopathy.^11^,^15^ These differences propose that the type of EPTB may be specific to adolescents in different population; more population-based studies in different geographic regions are required.

Approximately fifty percent of our adolescents were exposed to a known adult with TB. This finding shows that contact investigation remains an essential tool for the control of TB disease. Previous contact investigation may have prevented these cases of adolescents TB. Positive source cases were identified in 25–66% of the children with TB in previous studies.^16^–^19^ In our study, the rate of exposure to known adults with TB was close to the rate of children with TB. According to some studies, the history of contact with TB can be determined in 12%–19% of adult patients.^20^–^22^

Similar to the study conducted in the US, we found that adolescents aged 15 to 18 years were more likely to have smear-positive TB and severity of the disease.^23^ An epidemiological study on age and pathogenesis of TB has revealed that older adolescents are at higher risk of developing the disease than younger adolescents after infection with M. TB.^24^ Since most of these age groups have considerable social connections and interactions in colleges or schools; these patients are more likely to transmit TB to the community. Therefore, it is important to screen older adolescents for TB. Studies in children have revealed that the yields of culture and PCR are higher than that of the smear.^25^ Nevertheless, we found that the rate of smear positivity was higher than that of culture and PCR. Smear positive/culture negative results may be due to the presence of nonviable mycobacteria in the sample receiving anti-TB treatment. The low yields of culture and PCR might be due to low sample volume. More effort is needed to improve both quality and quantity of samples to have a better diagnosis of TB in adolescents. 26

Lymphadenopathy was the most common radiographic feature in young children.^27^ After puberty, the radiological findings of TB are similar to adults include parenchymal lesions and cavities.^28^ In the present study, the most common radiologic finding was infiltration, which is similar to studies conducted in the United States and Brazil.^11^,^29^ In contrast to the study conducted in France, most patients had mediastinal lymphadenopathy. However, despite this finding, a notable number of patients continue to present with a parenchymal lesion in France study.^12^ Although not statistically significant, cavitary lesion rate increased with age in our study. The studies on adolescent TB in France,^12^ Brazil^21^ and South Africa^15^ found that the cavity lesion rate increased with age. Seventy-one percent (n=101) of our patients were probably preventable through screening immigrants from Afghanistan (64) and contact investigation (37). Our study was consistent with the study performed in the US.^11^ This finding demonstrates the importance of developing new strategies for the prevention and treatment of TB to improve children’s and adolescents’ survival to achieve Millennium Development Goals.^30^

TB patients who are lost to follow-up are at increased risk of developing drug-resistant TB and treatment failure that further increases the risk of TB transmission.^31^ In this study, 54 patients (37.7%) were lost to follow-up after starting treatment. It is necessary to identify the components of potential behavioral intervention strategies for future program implementation among adolescents entering and receiving TB care. Contracting the contingency and peer counseling are two such intervention methods that have been used in adolescents.^32^ Peer counseling is an adolescent with a successful TB treatment encouraging a newly diagnosed adolescent patient. Contingency contracting is improving the desired behavior of adherence to the medical regimen by providing incentives.^33^ It has been suggested that prospective studies are required to evaluate such strategies to increase adherence to TB treatment.

Patients with EPTB were more likely to become lost to follow-up than those with PTB in this study. This result is in agreement with findings in previous studies.^34^,^35^ However, other studies have found no relationship between the type of TB and the successful TB treatment outcome.^36^–^38^ Despite the increasing proportion of patients with EPTB in many countries, these patients often receive less priority on the international health system.^39^ Therefore, more attention must be paid to the patients with EPTB, who are usually neglected in the TB control program.

Directly observed treatments (DOTs) should be attended to improve treatment compliance. There is a need to conduct prospective studies on the risk factors of loss to follow-up in adolescents TB treatment such as inadequate knowledge about TB, living far from health facilities, and drug side effects. Only one recent study revealed that the lowest rates of completing TB treatment were associated with older adolescent age, ethnicity, parental responsibility, and access to the clinic.^40^

## Conclusions

The study has shown that the clinical and radiological findings of TB in adolescents are a combination as identified in children and adults. The data presented in this study have implications for the development of strategies for early screening of TB in high-risk categories, prompt diagnosis of TB, and improving the rate of completion of care among adolescents treated for TB.

## Figures and Tables

**Table 1:** Evaluation of definite and probable TB in adolescents

		Definite TB N° (%)	Probable TB N° (%)
All		115 (80.4)	28 (19.6)
Age Groups years	10–14	23 (20)	17(60.7)
	15–18	92 (80)	11 (39.3)
Sex/male		39 (34)	14 (50)
Symptomatic		115 (100)	23 (95.7)
Contact with TB Patients		49 (42.6)	19(79.1)
TST	<10 mm	24 (20)	5 (20)
Status	≥10 mm	17 (14.7)	15 (62)
Smear positive		96 (83)	0
Culture positive		55/96 (57)	0
PCR positive		52/69 (75)	0
Histology positive		21	0
Radiology		81/85 (95)	21/24 (87)

**Table 2A:** Comparison of Adolescents with TB in two age groups

Variable		Total N=143 (%)	10–14 years N=40(%)	15–18 years N=103 (%)	P value
Gender	Female	90(62.9)	27(67.5)	63(61.2)	0.481
	Male	53(37.1)	13(32.5)	40(38.8)	
Symptomatic	Yes	138(96.5)	37(92.5)	101(98)	0.133
	No	5(3.5)	3(7.5)	2(2)	
Cough	Yes	123(86)	34(85)	89(86.4)	0.828
	No	20(14)	6(15)	14(13.6)	
Fever	Yes	104(72.7)	25(62.5)	79(76.7)	0.087
	No	39(27.3)	15(37.5)	24(23.3)	
Weight loss	Yes	94(65.7)	18(45)	76(73.8)	0.001
	No	49(34.3)	22(55)	27(26.2)	
TST Status	<10 mm	18(12.5)	13(39.3)	6(21.4)	0.167
	≥10 mm	32(22.3)	20(60.6)	12(42.8)	

**Table 2B:** Comparison of Adolescents with TB in two age groups

Variable			Total N=143 (%)	10–14 years N=40(%)	15–18 years N=103 (%)	P value
Microbiology	Smear	Positive	96(67.1)	18(45)	78(75.7)	0.001
		Negative	46(32.2)	22(55)	24(28.3)	
		Unknown	1(0.7)	0	1(1)	
	Culture	Positive	55(38.5)	9(22.5)	46(44.6)	<0.001
		Negative	68(47.5)	30(75)	38(37)	
		Unknown	20(14)	1(2.5)	19(18.4)	
	PCR	Positive	52(36.4)	13(32.5)	39(38)	0.009
		Negative	36(25.2)	17(42.5)	19(18.4)	
		Unknown	55(38.5)	10(25)	45(43.6)	
Radiologic Findings	Infiltration	Yes	62(43.4)	15(37.5)	47(45.6)	0.605
		No	80(55.9)	25(62.5)	55(53.4)	
		Unknown	1(0.7)	0	1(1)	
	Cavity	Yes	30(21)	8(20)	22(21.3)	1
		No	112(78.3)	32(80)	80(77.6)	
		Unknown	1(0.7)	0	1(1)	
	Adenopathy	Yes	23(16.1)	6(15)	17(16.5)	1
		No	119(83.2)	34(85)	85(82.5)	
		Unknown	1(0.7)	0	1(1)	

**Table 3:** Individual Characteristics of Adolescents for Follow-up TB Treatment

		Non-loss to follow-up TB treatment	Loss to follow-up TB treatment	P Value
All		89	54	
Age Groups	10–14 **years**	28	12	0.233
	15–18 **years**	61	42	
Gender	Female	57	33	0.725
	Male	32	21	
Ethnicity	Iranian	53	26	0.184
	Afghan	36	28	
Previous TB	Known	13	7	0.784
	Not known or unknown	76	47	
TB Type	Pulmonary	77	36	0.017
	Extrapulmonary	8	13	
	Both	4	5	
AFB Smear	Positive	65	33	0.087
	Negative or Unknown	24	22	
Presence of Cavitation	Yes	21	9	0.324
	No	68	45	
